# Predicting Neurodegenerative Disease Using Prepathology Gut Microbiota Composition: a Longitudinal Study in Mice Modeling Alzheimer’s Disease Pathologies

**DOI:** 10.1128/spectrum.03458-22

**Published:** 2023-03-06

**Authors:** Emily M. Borsom, Kathryn Conn, Christopher R. Keefe, Chloe Herman, Gabrielle M. Orsini, Allyson H. Hirsch, Melanie Palma Avila, George Testo, Sierra A. Jaramillo, Evan Bolyen, Keehoon Lee, J. Gregory Caporaso, Emily K. Cope

**Affiliations:** a Center for Applied Microbiome Sciences, the Pathogen and Microbiome Institute, Department of Biological Sciences, Northern Arizona University, Flagstaff, Arizona, USA; University of Nebraska—Lincoln

**Keywords:** Alzheimer’s disease, gut microbiome, gut-brain axis, *Bacteroides*

## Abstract

The gut microbiota-brain axis is suspected to contribute to the development of Alzheimer’s disease (AD), a neurodegenerative disease characterized by amyloid-β plaque deposition, neurofibrillary tangles, and neuroinflammation. To evaluate the role of the gut microbiota-brain axis in AD, we characterized the gut microbiota of female 3xTg-AD mice modeling amyloidosis and tauopathy and wild-type (WT) genetic controls. Fecal samples were collected fortnightly from 4 to 52 weeks, and the V4 region of the 16S rRNA gene was amplified and sequenced on an Illumina MiSeq. RNA was extracted from the colon and hippocampus, converted to cDNA, and used to measure immune gene expression using reverse transcriptase quantitative PCR (RT-qPCR). Diversity metrics were calculated using QIIME2, and a random forest classifier was applied to predict bacterial features that are important in predicting mouse genotype. Gene expression of glial fibrillary acidic protein (GFAP; indicating astrocytosis) was elevated in the colon at 24 weeks. Markers of Th1 inflammation (il6) and microgliosis (mrc1) were elevated in the hippocampus. Gut microbiota were compositionally distinct early in life between 3xTg-AD mice and WT mice (permutational multivariate analysis of variance [PERMANOVA], 8 weeks, *P* = 0.001, 24 weeks, *P* = 0.039, and 52 weeks, *P* = 0.058). Mouse genotypes were correctly predicted 90 to 100% of the time using fecal microbiome composition. Finally, we show that the relative abundance of Bacteroides species increased over time in 3xTg-AD mice. Taken together, we demonstrate that changes in bacterial gut microbiota composition at prepathology time points are predictive of the development of AD pathologies.

**IMPORTANCE** Recent studies have demonstrated alterations in the gut microbiota composition in mice modeling Alzheimer’s disease (AD) pathologies; however, these studies have only included up to 4 time points. Our study is the first of its kind to characterize the gut microbiota of a transgenic AD mouse model, fortnightly, from 4 weeks of age to 52 weeks of age, to quantify the temporal dynamics in the microbial composition that correlate with the development of disease pathologies and host immune gene expression. In this study, we observed temporal changes in the relative abundances of specific microbial taxa, including the genus Bacteroides, that may play a central role in disease progression and the severity of pathologies. The ability to use features of the microbiota to discriminate between mice modeling AD and wild-type mice at prepathology time points indicates a potential role of the gut microbiota as a risk or protective factor in AD.

## INTRODUCTION

The human microbiota, the aggregate of all bacterial, viral, fungal, and archaeal cells that inhabit the human body, consists of 1 to 1.5× more microbial cells than human cells (~10^14^) (1). Niche-specific microbiota reside across virtually the entire human body, including the skin, oral cavity, respiratory tract, vaginal cavity, and GI tract (2). The gut microbiota, which makes up approximately 70% of the total microbial burden in the body (3), contributes to a myriad of roles, including host immune regulation (4), macronutrient metabolism (5), and maintenance of overall health (6). In healthy individuals, the gut microbiota tends to be highly diverse (7). However, perturbations to the healthy gut microbiota caused by disease, aging, diet, or other environmental factors can lead to alterations in the composition or function of these communities. Alterations in a healthy gut microbiota are associated with inflammation and chronic noncommunicable diseases, such as obesity (8), diabetes (9), asthma (10), and inflammatory bowel disease (11–13). Recent studies of gut microbiota-associated effects of host health are beginning to demonstrate effects on extragastric organs, including neurological health and disease (14–16).

The gut microbiota-brain axis is the bidirectional communication between the gut and brain through immune, nervous, metabolic, and endocrine signaling (17). These collective mechanisms regulate a number of physiological processes, including gut motility and permeability (18), local and systemic inflammation (19), and normal brain function (20). Major perturbations to the gut microbiota-brain axis signaling are associated with diseases affecting the gastrointestinal tract, including Crohn’s disease (21) and irritable bowel syndrome (22), as well as the brain, including Parkinson’s disease (23), Alzheimer’s disease (AD) (24), autism spectrum disorder (25), and multiple sclerosis (26).

Alzheimer’s disease (AD) is an irreversible neurodegenerative disease characterized by the deposition of amyloid-β (Aβ) plaques and formation of neurofibrillary tangles in the brain, resulting in irreversible progressive memory loss. Patients with AD experience cognitive decline, often accompanied by anger, depression, and personality changes. Unfortunately, once symptoms become apparent, the individual will continue to decline until they are unable to perform daily tasks and communicate, and the disease is ultimately fatal (27). AD rates are rapidly increasing as our elderly population grows, with projections that cases will more than triple in the next 30 years (28). With no cure, and few therapies available to slow the progression, understanding disease pathogenesis is critical in the timely development of effective therapies. Currently, the main targets of AD therapies are neurotransmitter receptors, secretase inhibitors, modulation of amyloidosis and tauopathy, and immunotherapy (29). The Aβ-cascade hypothesis, which proposes that neurotoxic Aβ plaques are the causative agent of AD, leading to the formation of neurofibrillary tangles, vascular damage, and dementia, has more recently been brought into question, with increasing evidence against the long-standing hypothesis (30). Neuroinflammation has become a key research focus for AD, as it contributes to an increased rate of disease progression and severity (31).

Neuroinflammation in AD is characterized by a complex set of pathways, including dysfunctional microglia and astrocytes. Microglia are the resident macrophages of the central nervous system, while astrocytes function to support neuronal synaptic function and maintain the integrity of the blood brain barrier (BBB) (32, 33). Microglia clear soluble amyloid-β via macropinocytosis; however, in the insoluble, fibrillary form, microglia are unable to clear amyloid-β deposits at the rate they are forming, leading to the accumulation of amyloid-β plaques (34). The chronic neuroinflammation in AD is further characterized by proinflammatory biochemical processes, including the release of proinflammatory cytokines, mainly interleukin-1β (IL-1β), tumor necrosis factor alpha (TNF-α), and IL-6 (35). With mounting evidence of the role of neuroinflammation in AD pathogenesis, identifying shifts in inflammatory biomarkers during disease progression is increasingly important for identifying mechanistic pathways in the gut microbiota-brain axis.

In this study, we characterized the gut microbiota fortnightly through 52 weeks of age in 3xTg-AD mice with mutations associated with familial AD [APP(Swe), PSEN1(M146V) (bearing a change of M to V at position 146), and MAPT(P301L)], modeling amyloid-β plaques and hyperphosphorylated tau and their genetic background (B6129F2/J) (wild type [WT]). The APP(Swe) mutation in the amyloid precursor protein increases total amyloid-β, while the PSEN1(M146V) mutation of the cleavage enzyme induces abnormal APP processing, resulting in increased Aβ plaque accumulation (36). The third mutation, MAPT(P301L), accelerates the formation of neurofibrillary tangles (37). In this preclinical model, cognitive deficits develop at 4 months, preceding plaque accumulation at 6 months, gliosis at 7 months, and hyperphosphorylated tau at 12 months (38, 39). To our knowledge, this is the first study of its kind to characterize the gut microbiota composition of a transgenic AD murine model at 25 time points to identify key temporal patterns in the gut bacterial microbiome. Additionally, we compared changes in the gut microbiota composition to gene expression of key markers of inflammation using reverse transcriptase quantitative PCR (RT-qPCR). We hypothesized that alterations in the gut microbiome would correspond with key time points associated with the emergence of amyloid-β plaques, hyperphosphorylated tau, and neuroinflammation.

## RESULTS

### Longitudinal analysis of gut microbiota composition and inflammatory gene expression in 3xTg-AD mice.

**FIG 1:**
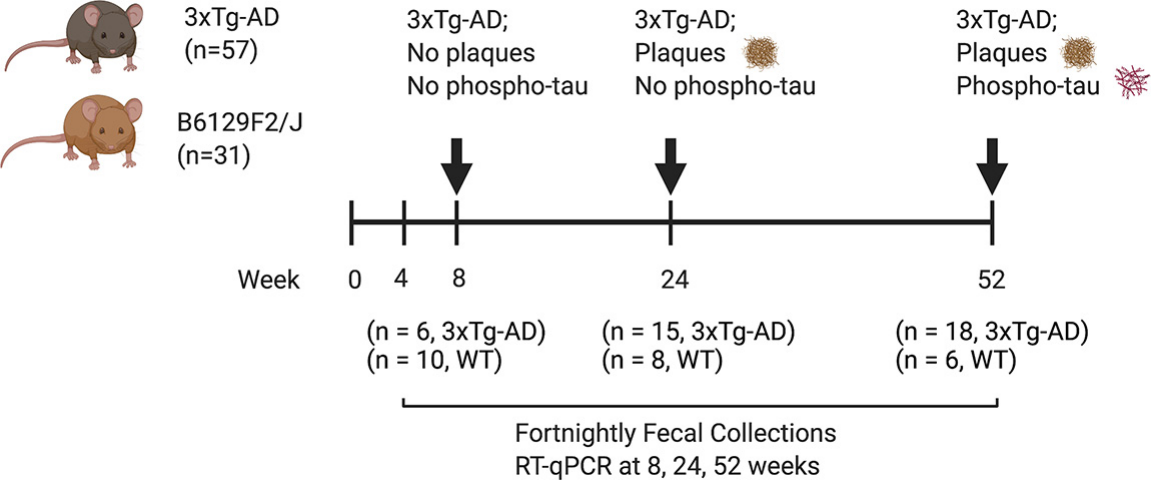
Longitudinal study design. Fecal sample collections from 3xTg-AD and WT mice began at 4 weeks of age and continued fortnightly until sacrifice at 8 weeks (prepathologies), 24 weeks (amyloidosis), and 52 weeks (amyloidosis and tauopathy). Image created using BioRender.com.

To explore shifts in gut microbial communities during disease progression, we used 16S rRNA gene sequencing to characterize fortnightly fecal samples from 4 weeks (postweaning) to 52 weeks (amyloid-*β* plaques and hyperphosphorylated tau model) of age. Our cohort consisted of 57 3xTg-AD mice and 31 WT mice, sacrificed at 8, 24, and 52 weeks (*n* = 88 mice and *n* = 1,079 total fecal samples at 25 time points) (Fig. 1). The mean sequencing depth was 29,429, with a range of 1,902 to 301,197 sequences per sample, with 55,911,922 total sequences generated. All of the representative sequences generated were assigned taxonomy at a minimum of 70% confidence. This led to a total of 3,779 amplicon sequence variants (ASVs). For core metrics, we rarefied to 10,692 sequences/sample, and we excluded 136 samples as a result. Gene expression of AD-associated inflammatory biomarkers was assessed at 8, 24, and 52 weeks of age.

### Inflammatory gene biomarkers in the hippocampus and colon.

**FIG 2:**
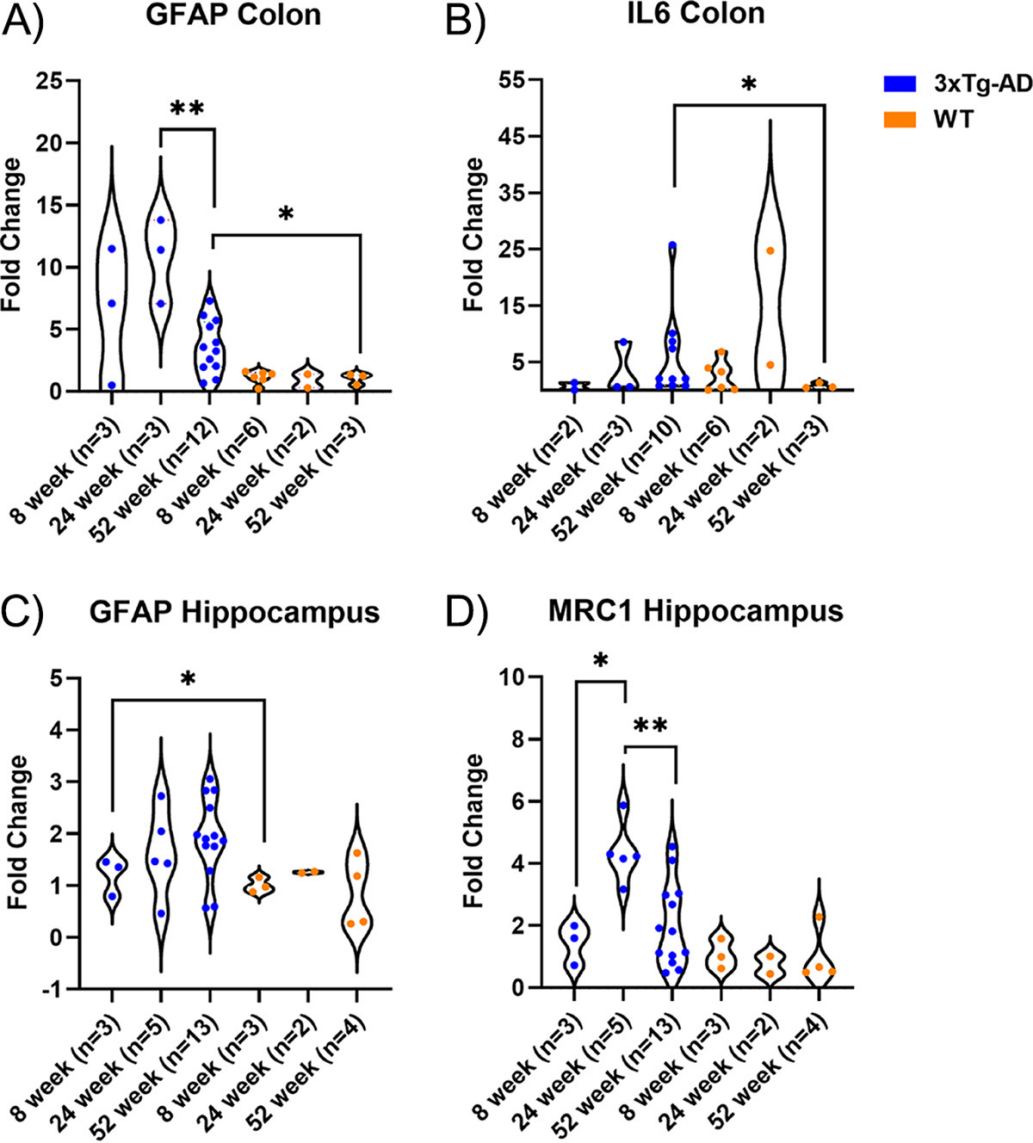
Relative levels of gene expression of GFAP and IL-6 in the colon and GFAP and MRC1 in the hippocampus. The hippocampus and colon from 3xTg-AD and WT mice were collected at 8, 24, and 52 weeks. (A) Gene expression of GFAP (astrogliosis marker) was significantly increased at 24 weeks in 3xTg-AD mice compared to 52 weeks in 3xTg-AD mice (*P* = 0.009, Mann-Whitney test) and was increased at 52 weeks in 3xTg-AD mice compared to 52 weeks in WT mice (*P* = 0.0484, Mann-Whitney test). (B) Gene expression of IL-6 was significantly increased at 52 weeks in 3xTg-AD mice compared to 52 weeks in WT mice (*P* = 0.015, Mann-Whitney test). (C) Gene expression of GFAP (astrogliosis marker) was significantly increased at 52 weeks in 3xTg-AD mice compared to 52 weeks in WT mice (*P* = 0.049, Mann-Whitney test). (D) Gene expression of Mrc1 (microgliosis marker) was significantly increased at 24 weeks in 3xTg-AD mice compared to 52 weeks (*P*= 0.004, Mann-Whitney test) and 8 weeks (*P*= 0.0357, Mann-Whitney test) in 3xTg-AD mice. *, *P* < 0.05; **, *P* < 0.01.

To assess the severity of the inflammatory response in the colon and hippocampus of 3xTg-AD mice, we used a custom reverse transcriptase qPCR assay to evaluate 24 genes for AD-associated inflammatory biomarkers. Based on previous characterization of pathologies in the brain of 3xTg-AD mice, the hippocampus was selected for neuroinflammatory marker analysis (40). Of the 19 genes assessed, 7 were T_H_1/T_H_17 markers, 3 were astrogliosis markers, 8 were microgliosis markers, 1 was a lipopolysaccharide (LPS)-induced inflammation marker, and 5 were controls/housekeeping genes (Table S1 in the supplemental material). Fold change values were calculated for hippocampus and colon samples from 8-, 24-, and 52-week-old 3xTg-AD and WT mice using the cycle threshold (2^−ΔΔ^*^CT^*) method. In the colon, the expression of the glial fibrillary acidic protein (GFAP; astrogliosis marker) gene was increased in 3xTg-AD mice at 24 weeks compared to its expression at 52 weeks (*P* = 0.009, Mann-Whitney test) (Fig. 2A), and IL-6 was increased in 3xTg-AD mice at 52 weeks compared to its expression in WT mice at 52 weeks (*P* = 0.049, Mann-Whitney test) (Fig. 2B). In the hippocampus, GFAP was increased in 52-week-old 3xTg-AD mice compared to the level in 52-week-old WT mice (*P* = 0.015, Mann-Whitney test) (Fig. 2C). The expression of Mrc1 (microgliosis marker) was also increased in the hippocampus of 3xTg-AD mice at 24 weeks compared to its expression at 52 weeks (*P*= 0.004, Mann-Whitney) (Fig. 2D).

### 3xTg-AD mice have a distinct gut microbiota composition prior to the development of AD-associated pathologies.

**FIG 3:**
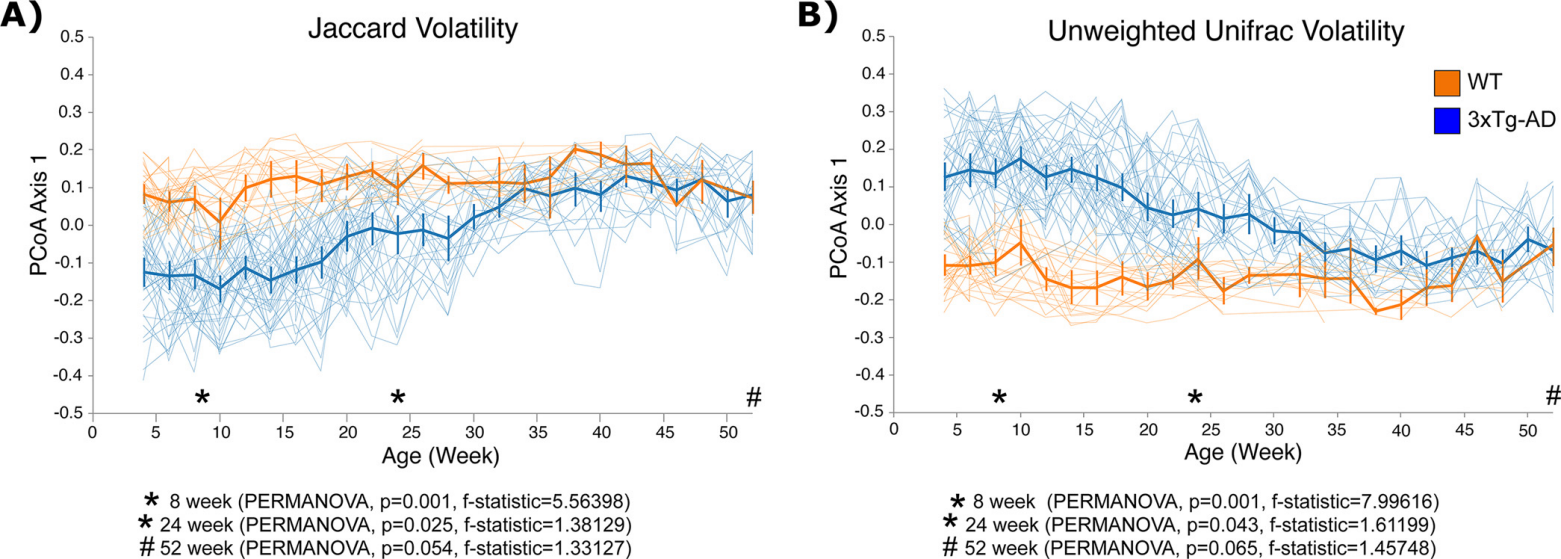
Volatility analysis of 3xTg-AD and WT mice from 4 to 52 weeks demonstrates distinct gut microbiota compositions in early life in 3xTg-AD mice compared to those in WT mice. (A) Volatility plot of PCoA axis 1 (PC1) of the Jaccard dissimilarity index. This demonstrates differences in the gut microbiota until 32 weeks of age by strain. Thick lines represent the average changes in the gut microbiota on PC1 over time in 3xTg-AD and WT mice, and thin lines represent changes in the gut microbiota on PC1 over time in individual mice. (B) Volatility plot of PCoA axis 1 (PC1) of unweighted UniFrac distance metric. This demonstrates differences in the gut microbiota until 42 weeks of age by strain. Thick lines represent the average changes in the gut microbiota on PC1 over time in 3xTg-AD and WT mice, and thin lines represent changes in the gut microbiota on PC1 over time in individual mice. Error bars show standard error.

Beta diversity (between-sample) metrics were used to identify compositional differences in the bacterial gut microbiota between 3xTg-AD and WT mice over time. We applied Jaccard and unweighted UniFrac, which are unweighted (qualitative) beta diversity metrics, and Bray-Curtis and weighted UniFrac, which are weighted (quantitative) beta diversity metrics, to our samples. Volatility analysis demonstrated a distinct gut microbiota composition for the first 30 weeks of age using Jaccard diversity (Fig. 3A) and 40 weeks using unweighted UniFrac (Fig. 3B) in 3xTg-AD mice compared to those in WT mice. As the mice aged, the compositions of the gut microbiota became more similar between the strains of mice (*n* = 88 mice and *n* = 1,079 total fecal samples at 25 time points) (Fig. 3). To analyze the differences in composition at 8 weeks (baseline), 24 weeks (when amyloid plaques are present), and 52 weeks (amyloid plaques and hyperphosphorylated tau were present), a principal-coordinate analysis (PCoA) of Jaccard and unweighted UniFrac distances was generated, with the first principal coordinate axis (PC1) plotted against time, highlighting the three key time points. The gut microbiota compositions were statistically distinct, using Jaccard and unweighted UniFrac metrics, between 3xTg-AD and WT mice in early life, shown at 8 and 24 weeks. However, the gut microbiota compositions became more similar at later time points, as demonstrated at 52 weeks of age (Fig. S3A, [PERMANOVA, *P* = 0.054, *F* statistic = 1.33127] and Fig. S3B, [PERMANOVA, *P* = 0.065, *F* statistic = 1.45748]). Notably, when we performed a multivariate PERMANOVA with genotype and cage as variables, we did see significant differences by genotype using both Jaccard (*P* = 0.037, *F* statistic = 1.412) (Table S2C) and unweighted UniFrac (*P* = 0.034, *F* statistic = 1.53) (Table S2F) distance metrics. This is discussed in further detail below.

**FIG 4:**
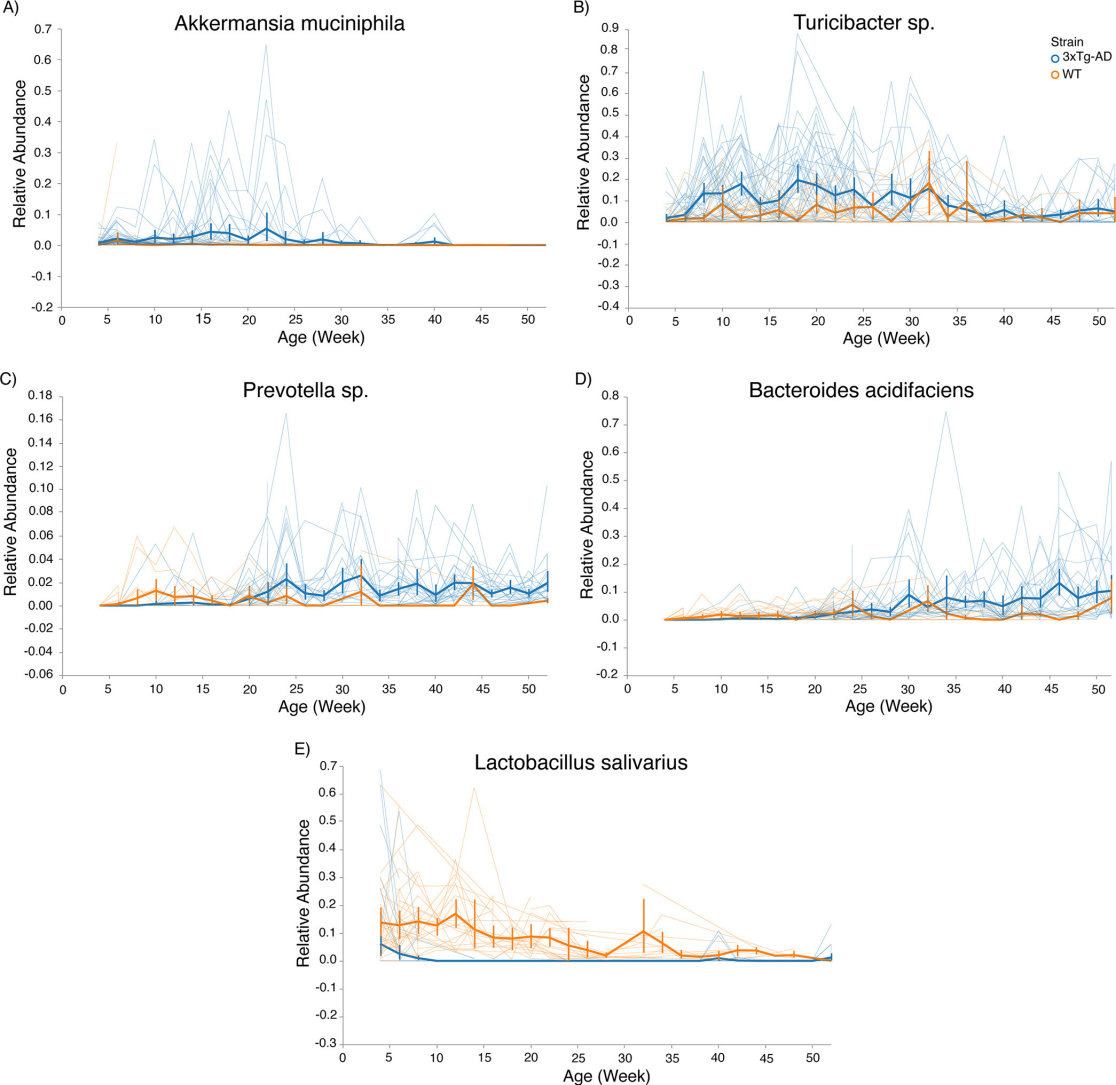
Feature volatility at species level. (A) Feature volatility chart of Akkermansia muciniphila demonstrates presence early in life in 3xTg-AD mice while being depleted in WT mice. (B) Feature volatility chart of Turicibacter species demonstrates an increase in relative abundance early in life in 3xTg-AD mice compared to WT mice and decreases over time in both mouse strains. (C) Feature volatility chart of Prevotella species demonstrates increasing abundance in 3xTg-AD mice after 20 weeks of age while being relatively stable in WT mice. (D) Feature volatility chart of Bacteroides acidifaciens demonstrates a stable increase in relative abundance in 3xTg-AD mice after 25 weeks of age. (E) Feature volatility chart of Lactobacillus salivarius shows depletion in 3xTg-AD mice compared to WT mice. Error bars show standard error.

Weighted beta diversity metrics showed a similar, though less robust pattern at the baseline and 24-week time points. Volatility analysis and PCoA of the Bray-Curtis dissimilarity metric demonstrated distinct gut microbiota compositions between 3xTg-AD and WT mice at 8 (PERMANOVA, *P* = 0.001, *F* statistic = 10.1743) and 24 (PERMANOVA, *P* = 0.016, *F* statistic = 1.98555) weeks of age, but not at 52 (PERMANOVA, *P* = 0.508, *F* statistic = 0.90456) weeks of age (Fig. S3A and C). A PCoA of the weighted UniFrac distance metric also demonstrated distinct gut microbiota compositions between 3xTg-AD and WT mice at 8 weeks (PERMANOVA, *P* = 0.03, *F* statistic = 3.10426) but not at 24 (PERMANOVA, *P* = 0.566, *F* statistic = 0.717805) or 52 (PERMANOVA, *P* = 0.066) weeks of age (Fig. S3B and D). Since weighted metrics did not demonstrate the strongest differences between genotypes, whereas unweighted metrics were highly significant, we interpreted the results as showing that the strongest drivers of different microbial communities were the lower-abundance taxa in the murine gut microbiota. This was supported by our volatility plots, where low-abundance taxa, such as Akkermansia (present at <10% relative abundance on average), Bacteroides (also present at an average of <10% relative abundance), Turicibacter (also present at average of <10% relative abundance), and Prevotella (present at <4% relative abundance), were among the most important features in the volatility analysis over time (Fig. 4).

In order to address differences in the gut microbiome due to cage and genotype, we performed multivariate PERMANOVA using the adonis function in QIIME2, which uses the adonis function in vegan-R (41). Not unexpectedly, larger percentages of variation in the gut microbiome at the three time points were due to cage effects; however, we still demonstrated that genotype contributed significantly to differences in the gut microbiome at each time point. Interestingly, using a univariate PERMANOVA, we did not observe significant differences in the unweighted metrics at 52 weeks of age. However, when we incorporated cage into the statistical model, we observed that there were significant differences in the gut microbiomes at 52 weeks (Jaccard, multivariate PERMANOVA, *P* = 0.037, *r*^2^ = 0.049, *F* statistic = 1.412; unweighted UniFrac, *P* = 0.034, *r*^2^ = 0.052, *F* statistic = 1.528) (Tables S2C and F). Overall, we demonstrated that, while cage accounted for an average of 37.7% of the variation (range = 32.3 to 42.0%) (Table S2), genotype remained significant at each time point and accounted for an average of 7% of the variation (range = 4.50 to 12.8%) (Table S2). We also performed volatility analysis on PC1, PC2, and PC3 based on Jaccard and unweighted UniFrac distance matrices. The goal was to determine which axes described clustering of the gut microbiota due to cage and genotype. We found that PC1, which described the greatest amount of variation (6%, Jaccard PC1; 11%, unweighted UniFrac PC1) (Fig. S4), described changes in genotype but not cage. Only PC3 seemed to show slight clustering by cage, and by definition, this principal coordinate described smaller variations in the gut microbiota (3%, Jaccard PC3; 4%, Unweighted UniFrac PC3) (Fig. S4). This supported our findings that genotype was a strong contributor to the gut microbiome composition. Furthermore, the contribution of genotype to variations in the gut microbiomes was akin to the findings of studies of the microbiome in chronic and progressive human disease, including the gut microbiome in neurological disorders (42–45).

### Bacterial features are differentially enriched in 3xTg-AD and WT mice over time.

To identify the ASVs that were driving the differences in gut microbiota compositions between 3xTg-AD and WT mice, feature volatility plots were produced using the QIIME2 plug-in q2-longitudinal. We identified five features at the species level. The longitudinal feature analysis demonstrated a temporal trend of increasing relative abundances of Akkermansia muciniphila and Turicibacter species early in life, while Prevotella species and Bacteroides acidifaciens increased after 24 weeks of age in 3xTg-AD mice. Furthermore, the longitudinal feature analysis identified Lactobacillus salivarius as an ASV that was depleted in 3xTg-AD mice.

Differential-abundance analysis of ASVs using analysis of the compositions of microbiomes (ANCOM) revealed a differential abundance of 59 ASVs between WT and 3xTg-AD mice at 8 weeks of age (Table S3). At 8 weeks of age, Akkermansia (W = 56, where W is defined as a count of the number of sub-hypotheses that pass for a specific taxon) and Turicibacter (W = 56) were differentially abundant ASVs enriched in 3xTg-AD mice, while Bacteroides (W = 57), Sutterella (W = 53), and Anaerostipes (W = 53) were enriched in WT mice. There were no differentially abundant taxa at 24 weeks, but at 52 weeks, 23 taxa were differentially abundant (Table S3).

### Associations between bacterial microbiota and mouse genotype over time using LME and random forest machine learning.

We applied a linear mixed effects (LME) model to determine the relationship of genotype (as a fixed effect) to gut microbiome diversity and Bacteroides acidifaciens abundance over time, leveraging the repeated measures for each mouse. When we performed pairwise comparisons at each time point, Faith’s phylogenetic diversity (Faith PD), an alpha diversity metric, was not significantly different in 3xTg-AD mice at 8 weeks compared to WT mice at 8 weeks (*P* = 0.098, Wilcoxon rank sum), 3xTg-AD mice at 24 weeks compared to WT mice at 24 weeks (*P* = 0.63, Wilcoxon rank sum), or 3xTg-AD mice at 52 weeks compared to WT mice at 52 weeks (*P* = 0.17, Wilcoxon rank sum) (Fig. S1). However, when we leveraged LME to analyze the effect of genotype on alpha diversity, Faith’s PD was significantly higher in the WT mice than in the 3xTg-AD mice at baseline (*P* < 0.001) and was consistently higher over time (*P* < 0.001). To evaluate the effect of genotype on microbial composition over time, we used LME on the first principal coordinate axis (PC1) from a PCoA generated from the Jaccard dissimilarity metric. The gut microbial composition of 3xTg-AD mice was significantly distinct at baseline (*P* < 0.001), and there were significant differences over time (*P* < 0.001). Genotype did interact with time in modulating the gut microbiome, suggesting a possible impact of genotype on microbiome development (*P* < 0.001). The gut microbiome composition changed more drastically from the baseline sample in 3xTg-AD mice compared to the gut microbiome in WT mice; the WT gut microbiome remained relatively stable over time. Finally, we wanted to determine whether there was a relationship between Bacteroides acidifaciens and genotype over time, since volatility analysis and ANCOM both demonstrated that this genus was enriched in 3xTg-AD mice. We applied LME to the relative abundances of Bacteroides acidifaciens, using genotype as a fixed effect. We demonstrated that the B. acidifaciens abundances at baseline were significantly different (*P* < 0.001) between 3xTg-AD and WT mice. Furthermore, we demonstrated differences in B. acidifaciens abundances by genotype (*P* = 0.049) and that there was a significant interaction between genotype and time (*P* < 0.001). These results demonstrated robust genotype-dependent changes in the gut microbiome over time.

We next used a random forest machine learning classifier to predict mouse genotype based on the bacterial features present in fecal samples. The random forest classifier was trained using 5-cross-fold cross validation on 80% of the samples and was then applied to the remaining 20% of samples to determine which taxa were most important in predicting mouse strain based on features of importance it identified in the training set. The feature tables were collapsed at the genus level and species level and combined with the feature table of ASVs prior to running the random forest classifier. We applied the random forest classifier to independent samples from the 8-week time point from all 57 mice to determine if gut microbiota features accurately predicted genotype, regardless of age. This time point was selected because it was prior to the onset of AD pathologies and because there was an adequate sample size to perform random forest analysis. At 8 weeks of age, random forest accurately predicted 3xTg-AD mice 88.9% of the time and WT mice 100% of the time, improving accuracy over the baseline by 1.4-fold (Table 1). Baseline accuracy was calculated by assuming every sample would be predicted as the metadata group with the largest sample size. Critically, these results demonstrated accurate prediction of genotype using a prepathology time point.

**TABLE 1:** Results of random forest sample classifier performed on young, prepathology 3xTg-AD and WT mice

Genotype	Age (wk)	Accuracy	Baseline ratio	Accuracy ratio
3xTg-AD	8	0.889	0.643	1.444
WT	8	1.00	0.643	1.444

## DISCUSSION

Despite numerous studies investigating how the gut microbiota is altered in AD, both in human and murine models, few studies have extensively sampled longitudinally to identify the dynamic gut microbiota signatures in 3xTg-AD mice. Previous studies have shown that 3xTg-AD mice have a distinct bacterial signature compared to age-matched controls (46–48). However, there are only two studies to our knowledge that have investigated gut microbiota in 3xTg-AD mice at more than one time point; the authors evaluated the gut microbiome at two and four (47, 49) time points. In one study, the gut microbiota of 3xTg-AD and WT mice were assessed at 8, 12, 18, and 24 weeks (49). They too demonstrated compositional differences that were highlighted at the 8-week time point, but the specific taxa that were depleted in the 3xTg-AD mice differed from those in our study. In the second study, the gut microbiota of 3xTg-AD and WT mice were assessed at 16 weeks and 24 weeks. They similarly demonstrated alterations in the gut microbiome prior to the development of pathologies, but they did not report taxonomic changes to the genus level (47). Here, we assessed the temporal dynamics by dense longitudinal sampling of microbial communities in the gut of 3xTg-AD mice over the course of a year to better understand compositional changes that correlate with disease pathologies. Our study characterized the gut microbiota compositions at 25 time points (*n* = 1,079 total samples), with multiple time points corresponding to prepathology development and plaque deposition and one time point corresponding to plaque deposition and hyperphosphorylated tau. Several bacteria, including Bacteroides acidifaciens, Prevotella species, Akkermansia muciniphila, Turicibacter species, and Lactobacillus salivarius, differed in their relative abundances between 3xTg-AD and WT mice over time. Turicibacter species and Akkermansia muciniphila were enriched in the gut microbiota in 3xTg-AD mice at early time points, preceding pathology development, while Bacteroides acidifaciens and Prevotella species were enriched in the gut microbiota of 3xTg-AD mice at later time points. Critically, these features in the gut microbiota were used to successfully predict the strain of mice early on in life, showing a potential for unique signatures in the gut microbiota composition to be used as a predictor of AD prior to the development of pathology.

Previous studies support the idea that perturbations in the gut microbiota composition alter host immune responses, thereby shifting toward a proinflammatory environment in the colon and hippocampus (50). To quantify changes in the inflammatory profile of 3xTg-AD mice, we assessed the expression of relevant neuroinflammatory and inflammatory genes at each body site. Significant increases in TNF-α, IL-6, IL-1β, and interferon gamma (IFN-γ) gene expression via RT-qPCR of brain tissue have been observed in 3xTg-AD mice at 16 months of age (51). In our study, we found significant upregulation of IL-6 gene expression in the colon of 52-week-old 3xTg-AD mice compared to its expression in 52-week-old WT mice, but no changes in TNF-α, IL-1β, and IFN-γ expression were observed at 52 weeks of age. We also observed significant upregulation of glial fibrillary acidic protein (GFAP), a marker of astrogliosis, in the hippocampus and colon of 3xTg-AD mice at 52 weeks compared to its expression in 52-week-old WT mice. Enteric glial cells (EGCs) are resident in the enteric nervous system, which aids in regulation of the gastrointestinal tract via modulation of immune and endocrine function (52). EGCs resemble astrocytes in the brain in their morphology, ability to secrete cytokines, and expression of glial fibrillary acidic protein. Increased gene expression of GFAP in the colon of rats 4 h after intravenous LPS injection suggests that GFAP upregulation is a result of acute exposure to a systemic inflammatory environment (52). Interestingly, GFAP has also been identified as a blood biomarker in AD patients and correlates with cognitive impairment (53). Finally, MRC1 (also known as CD206) was elevated in the hippocampus at 24 weeks of age in 3xTg-AD mice, indicating microgliosis. We hypothesize that the upregulation of MRC1 at 24 weeks of age is associated with increased phagocytosis in response to the deposition of amyloid-β, which is documented at 6 months of age (38).

In this study, we demonstrated distinct microbial compositions in 3xTg-AD mice prior to the development of AD pathologies. As the mice aged, the gut microbiota of 3xTg-AD and WT mice became more similar. Unweighted metrics (Jaccard and unweighted UniFrac) demonstrated significant differences at 8 and 24 weeks, but not at 52 weeks of age. We did observe significant differences using weighted beta diversity metrics (Bray-Curtis and weighted UniFrac), which accounted for the abundances of observed features at 8 weeks but not at 24 and 52 weeks. This indicated that lower-abundance bacterial microbiota features were strong drivers of changes in gut microbiota composition. Similar findings of compositional differences early in life were reported in female 3xTg-AD mice compared to B6129SF1/J mice at 3 and 5 months of age (47). Early-life gut microbiota composition perturbations in mice have been associated with aging-associated health and disease, including neurodegenerative diseases like AD (54). Our findings indicate that compositional differences in microbial communities, driven by rare taxa early in life, are present prior to amyloidosis and tauopathy development.

Alpha diversity is frequently used as a marker of disease status and is decreased in several diseases associated with the gut-microbiota brain axis, including depression (55), autism spectrum disorder (56), Parkinson’s disease (57), and in some studies, AD (16, 58). In humans, alpha diversity was reported to be decreased in elders with AD compared to the alpha diversity in age-matched healthy participants (16). When we analyzed alpha diversity metrics by subsampling our data to include one mouse at each time point, we did not find significant differences. These findings align well with other studies that have been performed in mice. In one, no differences in alpha diversity were reported when comparing 3- and 5-month-old 3xTg-AD female mice (47), and in another, no differences in alpha diversity were reported in 8-, 12-, 18-, and 24-week-old 3xTg-AD male mice compared to age-matched WT mice (49). However, when we leveraged dense longitudinal sampling using LME, we demonstrated that genotype had an effect on Faith’s PD, where WT mice had a higher alpha diversity than 3xTg-AD mice. These findings suggest that lower alpha diversity in 3xTg-AD mice may be a predictor of disease status when assessed during the onset and progression of AD pathologies.

To identify key features of the gut microbiota composition that differentiate 3xTg-AD mice from WT mice, we used a random forest machine learning classifier on a feature table of the fecal microbiota. Our analysis demonstrated successful discrimination between 3xTg-AD and WT mice using gut microbiota compositions from 4 to 52 weeks of age, but the prediction accuracy was improved when we included only samples from prepathology time points. We selected samples at 2 months of age (6 and 8 weeks) and 6 months of age (22 and 24 weeks) to increase sample size due to loss of samples during the sample classifier training. Several of the features that were most important for predicting strain were also significant in our other analyses, including Lactobacillus species, Lactobacillus salivarius, and Bacteroides species. The predictive power of these models indicates unique bacterial communities early in life and throughout life in 3xTg-AD mice modeling AD disease pathologies. Interestingly, Haran and colleagues were able to discriminate between elders with AD and elders with different types of dementia using a random forest model using strain-level features of the gut microbiome generated using shallow shotgun metagenomic sequencing (59). Both Bacteroides fragilis and Bacteroides vulgatus were important features in classifying participants in their study. Bacteroides species were also enriched in 3xTg-AD mice in our study. These findings suggest that certain microbes identified in the cohort with AD in this study, including Bacteroides species, may play a mechanistic role in the key pathologies of AD. We are performing additional studies to evaluate the role of Bacteroides in AD progression.

We observed concordance in the importance of features across our random forest classifier, longitudinal volatility analysis, and differential abundance testing (ANCOM). Analysis of feature volatility revealed taxa at the bacterial genus- and species-level resolution that are predictive of age within each strain. Prevotella species, associated with reductions in short-chain fatty acid production and intestinal inflammation in mice (60), were increased later in life in 3xTg-AD mice. Lactobacillus salivarius, a bacterium shown to positively influence immune cell development, was present in greater relative abundance in WT mice for the first 32 weeks of life (61). Akkermansia muciniphila, a mucin-degrading bacterium associated with intestinal inflammation in mice, and Turicibacter, a genus that can regulate intestinal serotonin production, were present in 3xTg-AD mice early in life, but not in WT mice (62, 63). Due to its potential influence on intestinal serotonin, Turicibacter is particularly interesting. This genus is also consistently identified in mouse and human studies of AD. One study using 5xFAD mice, which model amyloidosis at an earlier time point than 3xTg-AD mice, demonstrated increased relative abundances of Prevotella species, Bacteroides acidifaciens, and Turicibacter species in 5xFAD mice at 10 weeks of age (64). The 10-week time point in 5xFAD mice and the 24-week time point in 3xTg-AD mice each represent the development of amyloidosis in the respective models. This may indicate that changes in the relative abundances of certain microbes are critical during the onset of amyloid-β exposure. Another recent study of 5xFAD mice demonstrated that Turicibacter was depleted in 5xFAD mice at 18 months compared to its presence in WT controls (65). This study also demonstrated that Turicibacter was the most important feature of the gut microbiome differentiating 5xFAD and WT mice and may point to its importance in the gut microbiome-brain axis. Similarly, studies in humans find that Turicibacter is decreased in relative abundance in elderly patients with AD and age-matched controls (16). This could be a reflection of sampling late in the disease progression, since Turicibacter was enriched early in life in our study and was depleted at later time points (64, 65). Recently, Turicibacter species have been shown to contribute to the gut microbiome-brain axis via regulation of intestinal serotonin (5-HT) production, highlighting a potentially interesting mechanism for further study in AD, especially considering that serotonin potentially contributes to AD (63, 66, 67). These findings add to the exciting body of literature on the potential for specific microbial taxa to contribute to neurological health and disease.

All three statistical approaches used in our study (ANCOM, random forest machine learning, and volatility analysis) demonstrated increased relative abundance in Bacteroides in 3xTg-AD mice. Notably, random forest identified Bacteroides acidifaciens as highly important in predicting mouse strain. Other species of Bacteroides have been implicated in health status and are likely key contributors to host-microbial interactions via the gut microbiome-brain axis. Bacteroides fragilis and Bacteroides stercoris function ecologically as keystone species, indicated by low relative abundance and disproportionately numerous interactions in microbial community dynamics (68). B. fragilis can influence the gut microbiome-brain axis and reduce autism-like behaviors by modulating serum metabolites and GI inflammation (69). Bacteroides was also increased in abundance in mice expressing a variant of human APP (APPswe [Tg2576]) compared to its abundance in control mice, and the administration of B. fragilis promoted amyloid deposition in the APP/PS1 mice (70). These findings suggest the potential for amyloidosis to alter microbial communities in the gut of mice modeling AD amyloid-β plaques, or vice versa.

Bacteroides species have also been observed as differentially abundant in human studies of AD, though the associations with health or disease are conflicting. In one study of participants with AD and age-matched human controls, Vogt et al. demonstrated increased relative abundance of Bacteroides in patients with AD. Interestingly, this increase was positively correlated with a greater amyloid burden in the brain and cerebrospinal fluid (CSF) phospho-tau, indicating a greater disease burden (16). In another study, Haran and colleagues also observed increased Bacteroides in patients with dementia compared to age-matched controls (59). However, Zhuang et al. found that the relative abundance of Bacteroides decreased in patients with AD (71). Taken together, these findings in humans support our findings in a mouse model and suggest a role for gut-associated Bacteroides in the progression of AD pathologies.

Mechanistically, species in the genus Bacteroides might influence neuroinflammatory processes in the brain. Bacteroides fragilis produces an endotoxin, lipopolysaccharide, that is unique to this species of Gram-negative bacteria (BF-LPS). BF-LPS may cross the gut epithelium and enter the bloodstream, inducing systemic inflammation and upregulation of proinflammatory cytokines via the NF-κB pathway (72). BF-LPS is recognized by Toll-like receptor 2 (TLR-2), TLR-4, and CD41 microglial cells, potentially inciting microgliosis in the brain. We are currently investigating the role of B. acidifaciens in the ecology of the gut microbiota and hypothesize that it may also function as a keystone species and influence neurological health status through the gut microbiome-brain axis.

The complexity of the host-microbe interactions in 3xTg-AD mice was demonstrated in this study by the dynamic microbial communities and immune profiles. Our study characterized the gut microbiota temporally in 3xTg-AD mice modeling amyloid-β plaques and hyperphosphorylated tau to identify key changes in composition correlated with disease pathogenesis. The present study shows upregulation of biomarkers for microgliosis, astrogliosis, and intestinal inflammation. Analysis of the gut microbiome demonstrated an altered gut microbiota composition associated with 3xTg-AD early in life, including prior to pathology development, that was predictive of disease state. This is the first study of its kind to characterize the gut microbiota at 25 time points, ranging from prepathology to modeling of both amyloidosis and tauopathy. Additionally, it will provide a reference for future studies to determine the frequency of fecal sampling in longitudinal gut microbiota analysis based on the well-characterized evolving gut microbiota composition in the present study. It is critical for future studies on the role of the gut microbiota-brain axis and AD to investigate multiple time points throughout disease progression due to changes in the gut microbiome and inflammatory profile, as exemplified in the current study. Furthermore, a focus on the functional microbiome through a multiomics approach is essential in better understanding host-microbe interactions via the gut microbiota-brain axis in AD.

### Limitations and future directions.

While our study has strengths in dense, longitudinal sampling of fecal material, which allows for robust statistical analyses and modeling approaches, there are some limitations that warrant further discussion. First, after careful consideration of housing strategy, we chose to house genotypes separately at reduced density and to include multiple cages per experimental group at each time point, as suggested by Kim et al. (73). This choice was intentional, to eliminate the possibility of fecal microbiome transfer via coprophagy (and potentially transfer of disease phenotype or protection) between strains. Since our central hypothesis was that the gut microbiome influences disease progression in mice, we wanted to avoid the addition of potentially protective microbiota from WT mice to 3xTg-AD mice or the transfer of any disease phenotype from 3xTg-AD mice to WT mice. Some studies in 3xTg-AD (48) and 5xFAD (64) mice have demonstrated that cohousing mice across genotypes causes a shift in the gut microbiome so that the strains more closely resemble each other (expectedly), but this also alters the disease phenotype in transgenic and WT mice. In one study, cohousing young 3xTg-AD mice with aged 3xTg-AD mice accelerated AD pathology in the brain in young mice, demonstrating effective transfer of gut microbiome members by cohousing and the potential impact on pathologies. In 5xFAD mice, cohousing of WT and transgenic strains resulted in a shift of the WT gut microbiome to resemble the 5xFAD gut microbiome, reduced discriminatory learning, and resulted in an increase in brain-infiltrating T cells in WT mice compared to the level in WT mice in the same facility but caged separately (64). All this aside, we did not anticipate cage effects to be nonexistent. Thus, we performed multivariate analyses and volatility analyses to evaluate cage effects separately from genotype. These analyses support our findings that genotype is a significant contributor to gut microbiome differences between 3xTg-AD and WT mice. Furthermore, the contribution of genotype to variation in the gut microbiome is akin to studies of the microbiome in chronic and progressive human disease, including studies of the gut microbiome in neurological disorders (42–45).

There are additional factors that are challenging to control for in mouse studies. Cage effects are also confounded by maternal identity. This variable may be partially responsible for the larger percentage of variation accounted for by cage, since same-sex littermates were housed together. This could be one factor accounting for the larger variation in 3xTg-AD and WT mice at 8 weeks of age. However, it is important to note that while whole-community metrics (beta diversity) did converge over time (likely due to strict environmental controls, including exposure to the same bedding, chow, staff, etc.), we did see significance in the relative abundances of specific taxa at the 52-week time point, so key differences did remain. Furthermore, when we performed a multivariate PERMANOVA including cage and genotype as variables, we did observe significant differences in genotypes using beta diversity metrics.

We intentionally chose to use female mice in this initial study, which is the first out of our group exploring the gut microbiome alterations in AD. Our choice of female mice for this initial study was primarily driven by the finding that female sex is a leading risk factor for AD (74) and secondarily by the fact that female 3xTg-AD mice exhibit more consistent and greater pathology burden, whereas male 3xTg-AD mice have more variability in modeling key pathologies (75). Ongoing studies in our laboratory that build off these findings use both male and female mice. Studies on female mice in general are underrepresented in the literature, particularly in earlier studies of Alzheimer’s disease models (75, 76). The limitations discussed in the context of this study highlight the broader challenges in designing a study aimed at evaluating the role of the microbiome in a murine disease model and extend beyond AD research. There have been a few recent studies and commentaries on the challenges of murine study design, including husbandry, maternal effects, and diet (73, 77, 78), which were helpful in informing our study design. However, additional research toward mitigating these confounding factors is necessary, especially as the microbiome sciences advance toward understanding microbial mechanisms underlying disease pathologies.

## MATERIALS AND METHODS

### Mouse genotypes.

3xTg-AD [with overexpression of APP(Swe), PSEN1(M146V), and MAPT(P301L) transgenes] and wild-type (WT) (B6129F2/J) breeders were purchased from Jackson Laboratory (Bar Harbor, Maine). All mice included in this study were bred in-house at the Biological Sciences Vivarium at Northern Arizona University. All mouse experiments were approved by the Institutional Animal Use and Care Committee (IACUC) of Northern Arizona University under protocol 18-016, and we adhered to the IACUC regulations and animal housing conditions. All experiments and reporting were carried out in accordance with ARRIVE guidelines and regulations (79).

### Mouse colonies.

Mice were purchased from Jackson Laboratory (3xTg-AD and WT) and allowed 7 days to acclimate to the Animal Facility at Northern Arizona University. Mice were then combined into harems, housed in a 12-h light/dark cycle, and provided food and water *ad libitum*. In-house-bred mice were weaned at 21 days of age, and female mice of the same strain were separated and housed in cages of 2 to 5 mice (average of 4 mice/cage) for the remainder of the animal study (*n* = 88 total mice; *n* = 57 3xTg-AD and *n* = 31 WT). Multiple cages were used per experimental group at each time point, and cage was included as a variable in a multivariate PERMANOVA, described below. Weaned female mice were given 1 week to acclimate and adjust to their new food prior to the first sample collection.

### Genotyping.

Ear punches were collected at 4 weeks from 3xTg-AD mice for genotyping. DNA was extracted using the Qiagen blood and tissue kit (Qiagen, Hilden, Germany). PCR was run with the KAPA mouse genotyping kit and Jackson Laboratory-approved primers for APP(Swe) and MAPT(P301L) transgenes. Amplicons were run on a 3% agarose gel to confirm the presence of bands representing APP(Swe) and MAPT(P301L) genes (ThermoFisher, Waltham, Massachusetts).

### RT-qPCR.

DNA and RNA were extracted in parallel from hippocampus and colon tissue samples using the Qiagen AllPrep kit. We performed genomic DNA (gDNA) clean ups on RNA using the Qiagen DNase max kit. RNA was reverse transcribed using the Qiagen 2nd-strand synthesis kit (Qiagen, Hilden, Germany). A custom qPCR assay from Qiagen including various biomarkers for Th1/Th17 (*il2*, *il1beta*, *il6*, *il8*, *ifn-gamma*, *tnf-alpha*, and *il17a*), astrocyte reactivity (*GFAP*, *STAT3*, and VIM), M1/M2 macrophage activation/microgliosis (*ccl2*, *il1β*, *il4*, *arg1*, *iNOS*, *cd206*, *il10*, and *il12*) (80, 81), and LPS-induced neuroinflammation (NF-κB) were used.

### Sample collection.

Fecal samples were collected directly from each mouse fortnightly starting at 4 weeks until sacrifice for longitudinal gut microbiota analysis. Mice were euthanized with CO_2_ at 8, 24, or 52 weeks. Gastrointestinal and brain samples were collected in a sterile class II biosafety cabinet (BSC), using tools sterilized in a Germinator 500 (CellPoint Scientific), for each body site and mouse. The sample sizes were as follows: 3xTg-AD, *n* = 6 at 8 weeks, *n* = 15 at 24 weeks, and *n* = 18 at 52 weeks, and WT, *n* = 10 at 8 weeks, *n* = 8 at 24 weeks, and *n* = 6 at 52 weeks. The colon and hippocampus were harvested, immediately placed in RNAlater, and stored at −80°C until further processing.

### Nucleic acid extraction and 16S rRNA gene sequencing.

DNA and RNA were extracted in parallel from feces using the MagMax pathogen RNA/DNA kit from ThermoFisher. Extractions were performed in a class II biosafety cabinet using protocols adopted from eukaryotic cell culture to protect the samples from contamination (i.e., decontaminating all materials with 70% ethyl alcohol [EtOH] prior to bringing into the BSC, double gloving while in the BSC, and donning single-use personal protection equipment [PPE] while working in the BSC). Modifications to the protocol included the use of lysing matrix E tubes (MP Biomedical, Irvine, California) for bacterial and fungal lysis. Both DNA and RNA were quantified using a NanoDrop 2000. Quantified DNA from fecal samples was used for 16S rRNA gene PCR. Using Earth Microbiome Project (EMP) primers (515F-806R), the V4 region of the 16S rRNA gene was amplified. Each PCR mixture contained 2.5 μL of PCR buffer (10× concentration, 1× final; TaKaRa), 1 μL of the Golay barcode-tagged forward primer (10 μM concentration, 0.4 μM final), 1 μL of bovine serum albumin (20 mg/mL concentration, 0.56 mg/μL final; ThermoFisher), 2 μL of deoxynucleoside triphosphate (dNTP) mixture (2.5 mM concentration, 200 μM final; TaKaRa), 0.125 μL of hot-start *Ex Taq* (5 U/μL, 0.625 U/μL final; TaKaRa), 1 μL reverse primer (10 μM concentration, 0.4 μM final), and 1 μL of template DNA. All PCR mixtures were filled to a total of 25 μL with UltraPure DNase/Rnase-free water (Invitrogen) and then placed on a thermal cycler. The thermal cycler conditions were as follows: a 98°C denaturing step for 2 min, 30 cycles of 98°C for 20 s, 50°C for 30 s, and 72°C for 45 s, and a final step of 72°C for 10 min. PCR was performed in a decontaminated PCR hood, and consumables were decontaminated with 70% ethanol before being brought into the hood and then exposed to UV light to prevent sample contamination. PCR was performed in triplicate, and an additional negative control was included for each barcoded primer. 16S rRNA gene bands were visualized using a 3% agarose gel (ThermoFisher, Waltham, Massachusetts). Amplicons were quantified using fluorometry and pooled at equimolar ratios. The quality of the pool was assessed with the Bioanalyzer DNA 1000 chip (Agilent Technologies, Santa Clara, California), and the pool was then combined with 1% PhiX for sequencing. A total of 4 pools were sequenced on the Illumina MiSeq using the 600-cycle MiSeq reagent kit version 3 (Illumina, San Diego, California). Each pool contained mock communities and samples that overlapped over each sequencing run to identify potential sequencing bias. All sequencing was done on the Illumina MiSeq benchtop sequencing platform.

### Bioinformatics analysis.

Microbiome bioinformatics were performed with QIIME2 version 2021.2. A manifest of all commands used can be found in the supplemental material (cli_replay.sh and python3_replay.py). q2-DADA2 was used for sequence quality control and generation of amplicon sequence variants (ASVs) to provide the highest taxonomic specificity (82). A phylogenetic tree was created using q2-fragment-insertion, which applies the SEPP algorithm, inserting short sequences into a reliable tree generated from a database of full-length sequences (83). Taxonomy was assigned to reads using q2-feature-classifier and the Greengenes reference database, version 13_8 (83, 84). Alpha diversity, including Faith’s phylogenetic diversity (85), the Shannon diversity index (86), and observed ASVs, was computed with q2-diversity (85). Beta diversity (community dissimilarity) metrics were computed with q2-diversity, including Bray-Curtis dissimilarity, Jaccard dissimilarity, and weighted UniFrac (85, 87) and unweighted UniFrac (88) distances. Longitudinal analysis was performed with q2-longitudinal to assess temporal changes in bacterial communities (89). Group comparisons of alpha diversity were performed with nonparametric Wilcoxon tests, and group comparisons of beta diversity were performed with nonparametric PERMANOVA (90). Cage effects were assessed using volatility analysis with PC1 of Jaccard and unweighted UniFrac distances, and a multivariate PERMANOVA was performed using genotype and cage as covariates using adonis in R (41). ASVs and taxa that were differentially abundant across mouse strains were identified using ANCOM (91). All *P* values were corrected for multiple comparisons using the Benjamini-Hochberg false discovery rate correction. The random forest model sample classification was performed to predict mouse genotype using gut microbiome ASVs with q2-sample classifier (92).

### Statistical analysis.

Fold change values were calculated using the cycle threshold (2^−ΔΔ^*^CT^*) method (93). Group comparisons of strain and age were performed with the nonparametric Mann-Whitney test. Violin plots were created using Prism (GraphPad version 9.1.1.225).

### Data availability.

The data sets generated and/or analyzed during the current study are available in the NCBI Sequence Read Archive repository under accession numbers PRJNA830518 and PRJNA830532.
