# Supplementary material for: Predicting Neurodegenerative Disease Using Prepathology Gut Microbiota Composition: a Longitudinal Study in Mice Modeling Alzheimer’s Disease Pathologies
Source: Microbiol Spectr. 2023 Mar 6;11(2):e03458-22. doi: 10.1128/spectrum.03458-22 (PMC10101110; doi:10.1128/spectrum.03458-22)
Supplement: Supplemental file 1 — Supplemental material. Download spectrum.03458-22-s0001.pdf, PDF file, 2.8 MB [file spectrum.03458-22-s0001.pdf]

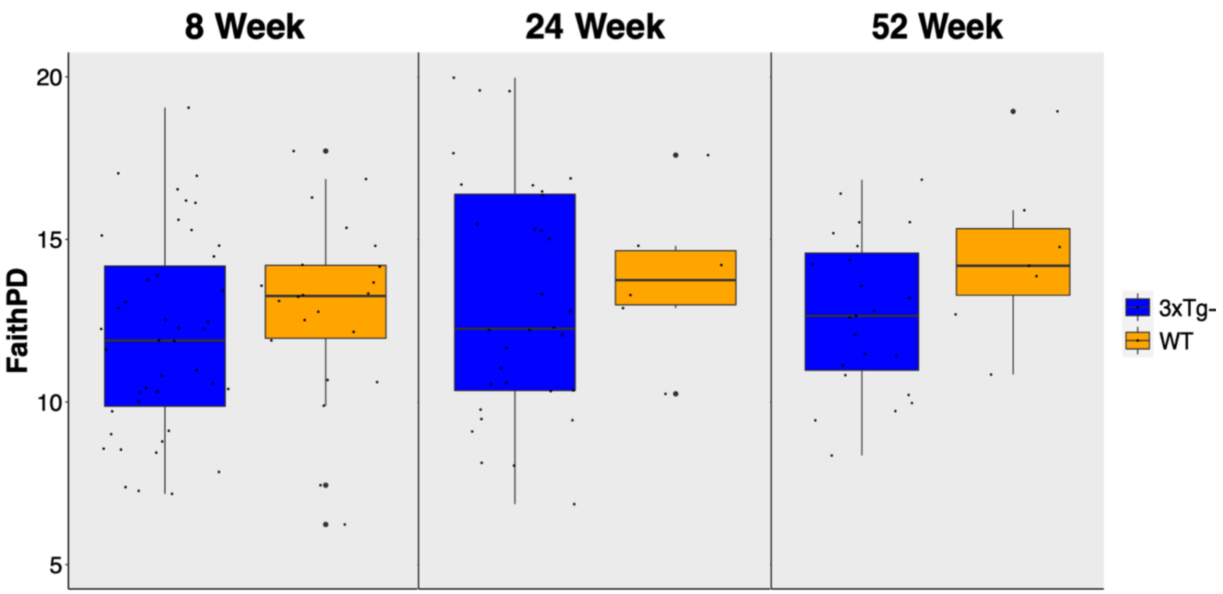

Figure S1: Faith's Phylogenetic Diversity in 8, 24, and 52 week 3xTg-AD and WT mice. 3xTg-AD mice demonstrate a non-significant trend towards lower Faith's Phylogenetic Diversity. A) 3xTg-AD and WT mice at 8 weeks (p-value = 0.098, Wilcoxon) B) 3xTg-AD and WT mice at 24 weeks (p-value = 0.63, Wilcoxon) C) 3xTg-AD and WT mice at 52 weeks (p-value = 0.17, Wilcoxon)

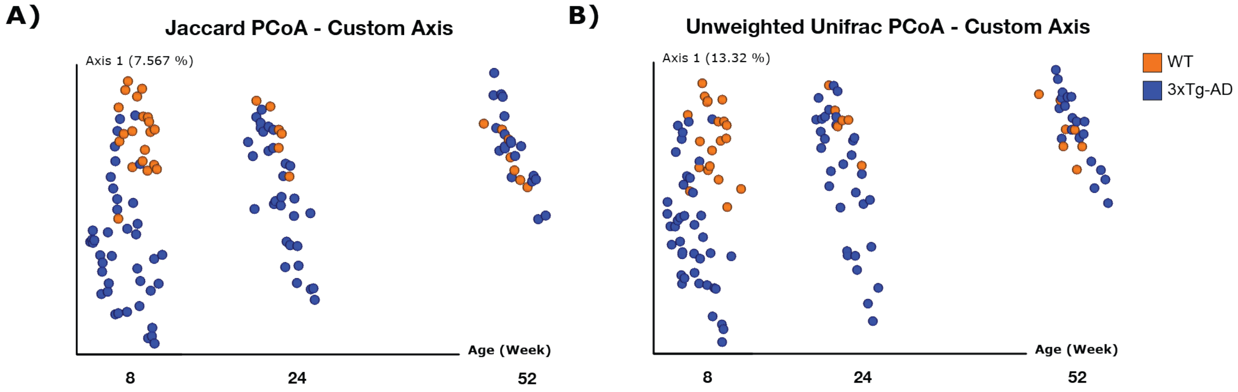

Figure S2: Jaccard dissimilarity metric and Unweighted Unifrac PCoA 1 plotted against time of 3xTg-AD and WT mice from 4 to 52 weeks demonstrate distinct gut microbiota

compositions in early life in 3xTg-AD mice compared to WT mice. A) PCoA of Jaccard dissimilarity metric, with key timepoints in pathology progression plotted as a PCoA 1 plotted against time (baseline: 8 weeks, amyloidosis: 24 weeks, tauopathy: 52 weeks). This demonstrates distinct gut microbiota compositions between 3xTg-AD and WT mice at 8 (PERMANOVA, p=0.001, f-statistic=5.56398) and 24 (PERMANOVA, p=0.025, f-statistic=1.38129) weeks of age, but not at 52 (PERMANOVA, p=0.054, f-statistic=1.33127) weeks of age B) PCoA of Unweighted UniFrac distance metric, with key timepoints in pathology progression plotted as a PCoA 1 plotted against time. This demonstrates distinct gut microbiota compositions between 3xTg-AD and WT mice at 8 (PERMANOVA, p=0.001, f-statistic=7.99616) and 24 (PERMANOVA, p=0.043, f-statistic=1.61199) weeks of age, but not at 52 (PERMANOVA, p=0.065, f-statistic=1.45748) weeks of age.

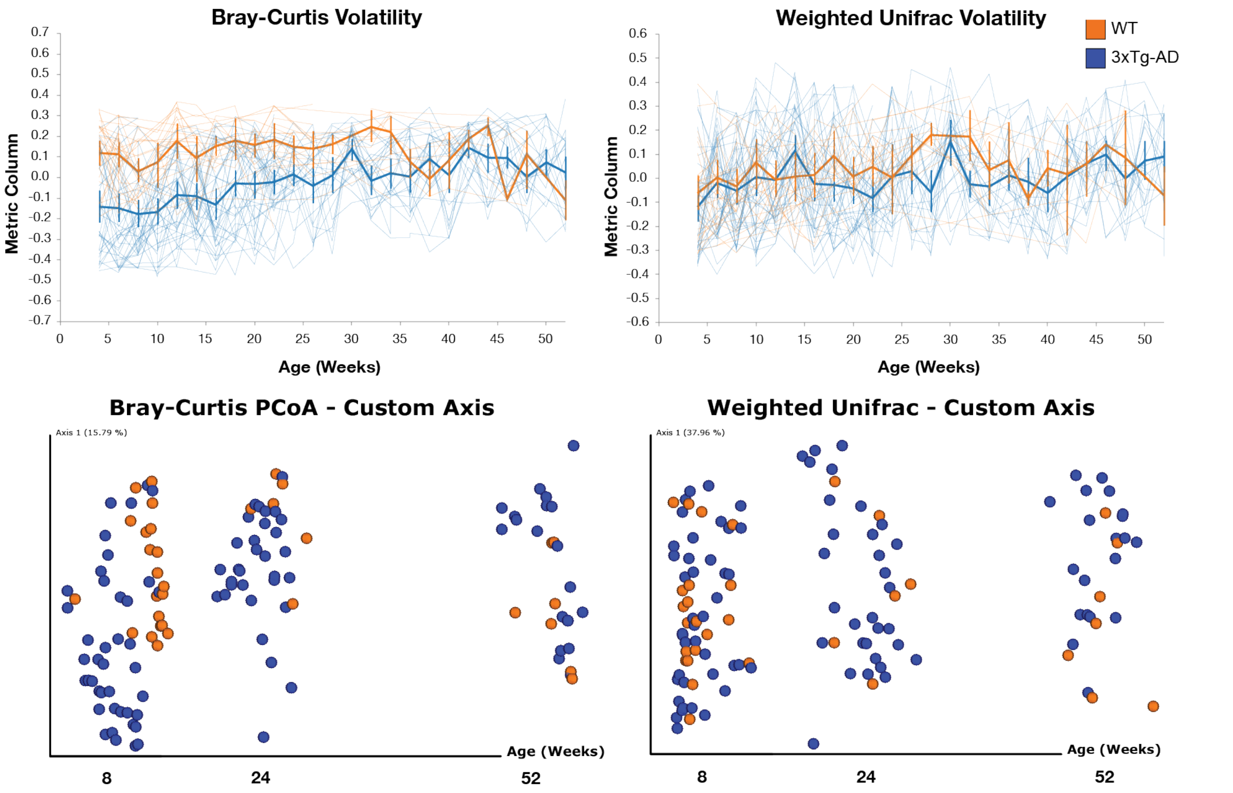

Figure S3: Beta-diversity metrics of 3xTg-AD and WT mice from 4 to 52 weeks of age and at 8, 24, and 52 weeks when comparing mouse strain. A) Bray-Curtis Axis 1 Volatility Plot from 4 to 52 weeks of age shows distinct gut microbiota compositions of 3xTg-AD and WT mice until 24 weeks of age. B) Weighted Unifrac Axis 1 Volatility Plot from 4 to 52 weeks of age. C) Bray-Curtis PCoA 1 plotted against time demonstrates distinct gut microbiota compositions between 3xTg-AD and WT mice at 8 (PERMANOVA, p=0.001, f-statistic=10.1743) and 24 (PERMANOVA, p=0.016, f-statistic=1.98555) weeks of age, but not at 52 (PERMANOVA, p=0.508, f-statistic=0.90456) weeks of age. D) Weighted Unifrac PCoA 1 plotted against time demonstrates distinct gut microbiota compositions between 3xTg-AD and WT mice at 8 (PERMANOVA, p=0.03, f-statistic=3.10426) , but not at 24 (PERMANOVA, p=0.566, f-statistic=0.717805) and 52 (PERMANOVA, p=0.066) weeks of age.

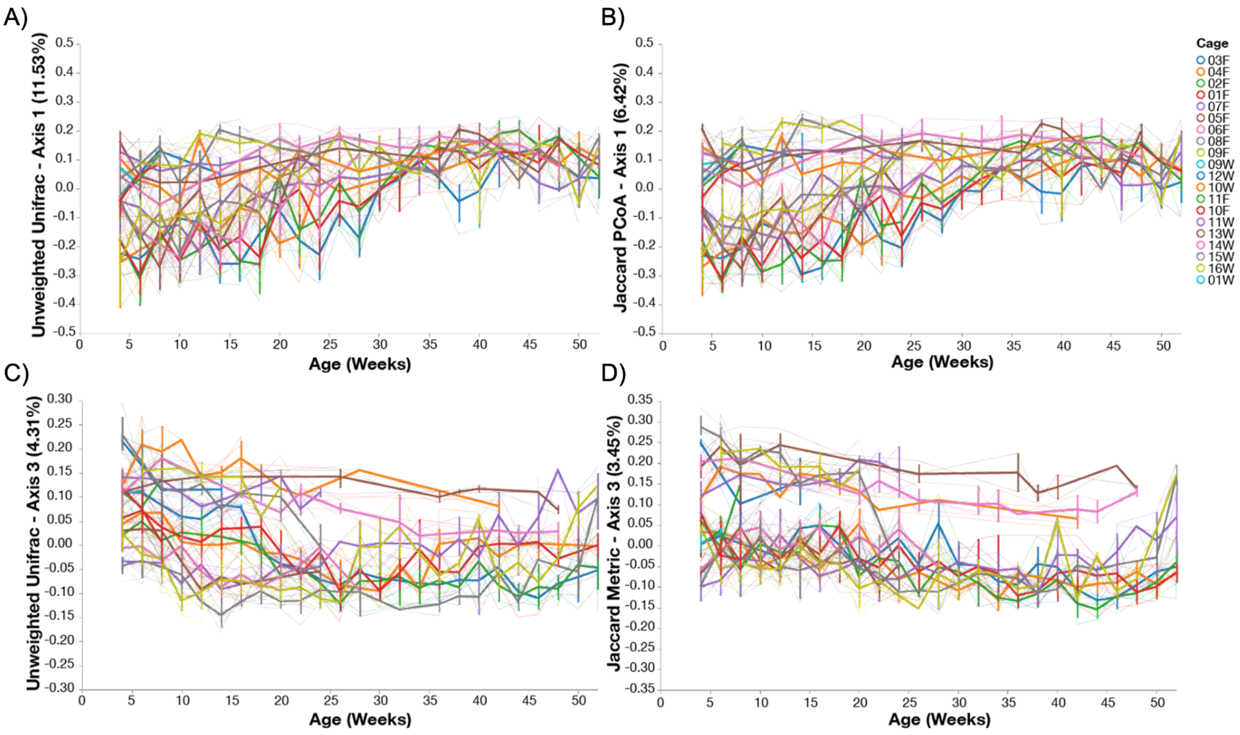

Figure S4. Volatility analysis colored by cage. Panels A) and B) demonstrate that cage is not the primary driver of gut microbiome composition since there is no visible clustering by cage on PCoA axis 1, which explains the greatest amount of variation. Panels C) and D) show clustering by cage on PC3, which explains the less variation.

Table S1: qPCR gene biomarkers included in the custom RT-qPCR assay

**Marker types**	**Genes**
Th1/Th17	*il2, il1beta, il-6, il-8, ifn-gamma, tnf-alpha, il17a*

astrocyte reactivity	*GFAP, STAT3, vimentin*
M1/M2 macrophage activation/microgliosis	*ccl2*, *il1β, il4, arg1, iNOS, cd206, il-10,* and *il-12, mrc1*
LPS-induced neuroinflammation	NF-kB

Table S2A: Jaccard 8 week multivariate analysis

	F Model	R2	p-value
**Genotype**	7.840421	0.091967	0.001
**Cage**	2.386569	0.391918	0.001
**Residuals**	NA	0.516115	NA
**Total**	NA	1	NA

Table S2B: Jaccard 24 week multivariate analysis

	F Model	R2	p-value
**Genotype**	1.891222	0.045374	0.001
**Cage**	1.973685	0.378819	0.001
**Residuals**	NA	0.575806	NA
**Total**	NA	1	NA

Table S2C: Jaccard 52 week multivariate analysis

	F Model	R2	p-value
**Genotype**	1.411924	0.049095	0.037
**Cage**	1.869423	0.325015	0.001
**Residuals**	NA	0.625890	NA
**Total**	NA	1	NA

Table S2D: Unweighted Unifrac 8 week multivariate analysis

	F Model	R2	p-value
**Genotype**	12.167799	0.127776	0.001
**Cage**	2.789998	0.410174	0.001
**Residuals**	NA	0.462050	NA
**Total**	NA	1	NA

Table S2E: Unweighted Unifrac 24 week multivariate analysis

	F Model	R2	p-value
**Genotype**	2.351515	0.051773	0.005
**Cage**	2.383485	0.419818	0.001
**Residuals**	NA	0.528409	NA

**Total**	NA	1	NA

Table S2F:
Unweighted Unifrac 52
week multivariate
analysis

	F Model	R2	p-value
**Genotype**	1.528134	0.051964	0.034
**Cage**	1.975922	0.335953	0.001
**Residuals**	NA	0.612084	NA
**Total**	NA	1	NA

Table S3: Differential abundance between 3xTg-AD and WT mice using Analysis of Composition of Microbiomes (ANCOM) at 8 weeks of age collapsed at genus level. W represents the number of features that the taxa is more abundant than.

Features Collapsed at Genus Level	W	Week
Bacteroides	57	8
Akkermansia	56	8
Turicibacter	56	8
Sutterella	53	8
Anaerostipes	53	8
F. Coriobacteriaceae	11	52
F. Mogibacteriaceae	9	52

Adlecreutzia	7	52
O. RF39	6	52
Prevotella	6	52
F. Erysipelotrichaceae	6	52
O. Streptophyta	6	52
Clostridium	5	52
Akkermansia	5	52
Bacillus	5	52
